# Annotations

**Published:** 1898-09-24

**Authors:** 


### ANNOTATIONS.

### Fresh Air for City Children.

It is unfortunate that the banks of London's great waterway have been to such a large extent devoted to the conduct of offensive trades that a trip down the river has become a somewhat doubtful pleasure. Still, we would often willingly exchange the heat and stifling odours of the streets for a seat under an awning on a steamer on the Thames, even though we should have to pass certain establishments from which smells anything but refreshing are apt to float over the water, and we feel sure that on many and many a day in the summer time London children would find infinite good, as well as untold pleasure, if there were provided for their use such a floating hospital as for twenty years past has been in the habit of carrying out each morning from New York its daily load of infants to breath the pure air which it is difficult for them to obtain in the tenements in which they dwell. On this ship are a few cots and beds for cases too ill to sit outside, but the great mass of the "patients" sit on deck, or play up and down, breathing fresh air and enjoying sea breezes. Then feeding time comes round, and both the children and the mothers—for no infants come without their mothers—get for once a good meal. Bathing is another great feature of these trips, and on the lower deck baths of various sorts are supplied, so that the little ones return after their outing with clean skins and full stomachs, with bodies revived by the sea air, and minds refreshed by new sights which they will not readily forget. This is a health-giving trip such as we, with the present surroundings of our river, can hardly hope to give. But even with our gasworks, our sewage works, our artificial manure factories, and other sweet appendages of civilisation, we can at least offer something better than the air of some of our courts and alleys. In any case a properly equipped steamer, with good provision for meals for the hungry and for rest for the weakly ones, with good sanitary arrangements and proper supervision by nurses all the time, would seem to be a better way of giving a whiff of fresh air to children of the slums than the ordinary "day in the country" or "day at the seaside," excursions which involve long, weary journeyings in stuffy railway carriages and exposure to all sorts of insanitary abominations at the journey's end.

### A Poor-Law Sanatorium.

Dr. Nathan Raw, medical superintendent of the Mill Road Poor Law Infirmary, Liverpool, is to be congratulated on the action he has taken in regard to the treatment of consumption among the poor. He has presented to the chairman and members of his committee a report on the open air treatment of tuberculosis in sanatoria, and has urged upon them the desirability of erecting a building to accommodate 30 or 40 patients and four nurses, where the treatment could be carried out. This is a matter which specially and urgently comes within the province of the guardians, for almost all the cases of consumption which occur among the really poor drift of necessity into the hands of the poor law authorities, and so far as their present arrangements go the treatment they receive can only be palliative. This is especially a case in which a stitch in time would save much misery and much loss, and we hope the guardians of Liverpool will give to Dr. Raw's proposal the careful consideration which it deserves. Two duties seem to lie upon poor law guardians in regard to tuberculosis. One is to provide asylums for those who are helplessly ill from consumption. To take care of them in some form is a thing they must do—it is part of their guardianship; and they are, we think, in honour bound to deal with their task in such a manner as not to cause injury to the other paupers. This duty, then, is one which is laid upon them by law, and from which they ought not to attempt to escape. The other is the provision of specially-equipped sanatoria for the treatment of the earlier cases, as suggested by Dr. Raw. This may, perhaps, be looked upon as a matter of policy rather than necessity; but the policy of undertaking the restorative treatment of a disease which, if not cured, will certainly throw the victim and probably some of his family for long years upon the rates, seems to us to be one which is so nearly allied to a duty as to be indistinguishable from it.

### The Use of Oxygen by Aeronauts.

The remarkable balloon ascent made last week by Mr. Stanley Spencer and Dr. Berson, when the great altitude of 27,500 ft. was attained, is interesting among other things for the fact that the respiration of oxygen was found of great service in relieving the oppression to the breathing which was caused by the extreme rarefaction of the air. The highest balloon ascent on record is that made by Messrs. Glaisher and Coxwell in 1862, when an altitude of upwards of 29,000 ft. is stated to have been reached. Before this height had been attained Mr. Glaisher lost consciousness, and Mr. Coxwell nearly became insensible. The exertion of breathing must itself be very considerable when the air is so rarefied, as we must at once recognise when we remember that at the height reached by Coxwell the barometer stood at only 7 in. as compared with 29 in. at the surface of the earth, so that each inspiration would give less than one-fourth the usual quantity of air, even without reckoning any diminished activity of gaseous exchange. It is hoped that by breathing oxygen instead of ordinary air it may be possible to reach still greater heights without interference with vital functions.

### Inflammable Hair Washes.

The annual report made by Mr. Spencer, chief officer of the Public Control Department of the London County Council, which was issued at the beginning of this month, states that a notice was issued some time ago pointing out the danger of using petroleum spirit for cleansing the hair, and was delivered to eighty-five hairdressers' premises; and it is further stated that the use of this dangerous liquid by hairdressers has ceased, so far as can be ascertained. It seems, however, that women have not yet entirely given up the use of inflammable hair washes, notwithstanding the warnings given by the terrible accidents which have occurred from their employment. Another death from this cause has lately taken place at Margate. From the report of the inquest we find that the deceased girl was accustomed to clean her hair with a benzoline preparation, and that on September 3rd, after so doing, her hair caught fire through her going near a lighted gas-stove. She was badly burnt, and died on the 7th inst.

